# Supplementary material for: ECG-Based Semi-Supervised Anomaly Detection for Early Detection and Monitoring of Epileptic Seizures
Source: Int J Environ Res Public Health. 2023 Mar 12;20(6):5000. doi: 10.3390/ijerph20065000 (PMC10049350; doi:10.3390/ijerph20065000)
Supplement: Supplementary file 1 [file ijerph-20-05000-s001.zip › ijerph-2201521-supplementary.pdf]

Supplementary Material:

S1. Model performance per patient

In Table S1. of our supplementary material we present the performance of the three studied anomaly detection methods, LOF, MCD and OCSVM on each seizure case contained in the PIHROPE dataset.

Table S1. A per case breakdown of model performance

Model	Case	Threshold	ΔT_W [2]	AUC (%)	Accuracy (%)	Sensitivity (%)	Specificity (%)
LOF	sz01-1	−2.66/−1.99	06:28/06:28	100.0/95.4	100.0/93.4	100.0/90.6	100.0/99.6
	sz02-1	−1.65/−2.53	29:02/28:26	99.7/99.2	96.5/94.7	94.7/94.3	100.0/95.2
	sz02-2	−1.51/−1.73	20:46/20:36	99.9/92.2	99.5/91.9	98.9/100.0	100.0/87.1
	sz03-1	−1.43/−1.46	21:16/21:16	94.3/96.8	90.4/91.2	84.2/89.2	94.5/92.2
	sz03-2	−1.47/−4.45	07:13/07:13	98.0/99.9	93.9/99.5	89.7/100.0	94.6/99.4
	sz04-1	−1.61/−1.78	05:58/05:58	96.4/99.9	88.8/97.9	97.3/99.5	83.2/96.6
	sz05-1	−1.18/−2.43	13:20/13:20	68.6/100.0	55.8/100.0	33.7/100.0	98.6/100.0
	sz06-1	−1.09/−1.03	06:58/06:58	90.5/88.8	86.8/73.9	76.2/86.7	91.8/69.3
	sz06-2	−1.37/−3.99	06:28/06:28	97.8/100.0	97.4/99.9	95.8/100.0	98.1/99.9
	sz07-1	−3.18/−3.81	22:58/22:53	100.0/99.7	100.0/99.4	100.0/100.0	99.9/98.9
AVG±STD	***Hand-picked***	−1.77±0.64	14:07±8:40	97.4±3.1	94.8±4.8	93.0±7.7	95.8±5.3
	Weak	−2.53±1.17	14:01±8:32	96.9±3.8	93.5±7.7	95.6±5.2	93.1±9.3
MCD	sz01-1	−2.50/−2.50	06:17/06:17	99.6/97.8	97.9/91.2	96.8/87.4	99.7/100.0
	sz02-1	−1.87/−1.96	28:52/26:28	97.0/96.9	86.2/90.6	78.8/86.2	99.9/95.5
	sz02-2	−1.82/−1.92	20:59/20:59	97.6/91.3	91.0/85.1	98.8/95.3	84.2/78.9
	sz03-1	−1.31/−1.31	21:31/21:31	88.2/87.6	79.3/77.0	83.6/85.6	76.4/72.4
	sz03-2	−1.71/−1.80	07:13/07:13	95.1/99.7	95.9/98.6	72.4/100.0	99.6/98.5
	sz04-1	−2.31/−2.35	05:58/05:58	84.3/95.4	82.1/91.4	91.1/93.7	76.1/89.5
	sz05-1	−0.87/−2.32	13:29/13:20	62.9/99.1	72.3/97.4	85.4/100.0	46.9/97.1
	sz06-1	−1.72/−1.84	06:58/06:58	75.3/70.8	67.7/71.3	85.6/69.8	59.2/71.8
	sz06-2	−2.07/−2.76	06:28/06:28	99.1/99.4	97.7/97.1	95.5/99.4	98.6/96.8
	sz07-1	−2.16/−2.44	22:58/22:30	87.1/87.5	83.5/83.5	99.2/89.9	69.6/78.1
AVG±STD	***Hand-picked***	−2.07±0.54	14:06±8:41	91.6±7.9	86.9±9.4	88.2±8.3	85.7±14.7
	Weak	−2.20±0.53	13:37±8:02	92.4±8.9	88.9±9.3	91.1±9.2	87.8±10.5
OCSVM	sz01-1	1.12/1.13	06:27/06:28	100.0/97.8	99.6/94.0	99.4/91.9	100.0/98.8
	sz02-1	1.93/1.85	28:54/28:41	98.3/98.3	95.1/95.2	92.5/99.9	100.0/89.9
	sz02-2	1.19/1.06	20:49/20:41	100.0/91.6	99.6/91.7	99.2/100.0	99.9/86.8
	sz03-1	2.07/2.00	21:21/21:17	96.0/98.7	91.0/95.2	91.2/90.8	90.8/97.5
	sz03-2	2.75/1.91	07:13/07:13	94.0/100.0	87.6/99.8	80.4/100.0	88.8/99.8
	sz04-1	1.50/1.35	05:58/05:58	96.7/99.9	90.0/98.5	100.0/100.0	83.3/97.2
	sz05-1	2.10/1.76	13:20/13:20	50.1/100.0	49.3/100.0	28.3/100.0	90.1/100.0
	sz06-1	1.83/1.67	06:58/06:44	81.7/74.4	79.2/86.6	77.6/49.2	79.9/99.9
	sz06-2	1.89/0.02	06:28/06:28	98.5/100.0	97.3/99.7	95.8/100.0	97.9/99.7

	sz07-1	2.36/2.20	22:40/22:35	99.9/100.0	99.4/100.0	98.6/100.0	100.0/100.0
AVG±STD	***Hand–picked***	1.85±0.50	14:05±8:38	96.1±5.5	93.2±6.5	92.7±7.9	93.4±7.5
	Weak	1.47±0.63	14:00±8:35	95.6±7.9	95.6±4.2	92.4±15.7	96.6±4.6

[1] Metrics format: Hand-picked label metrics / Weak label metrics. [2] Time format: Min: Sec
